# Experimental Research on Application of Waste Concrete Powder–Waste Brick Powder–Cement Grout for Foundation Reinforcement in Mining Goaf

**DOI:** 10.3390/ma16186075

**Published:** 2023-09-05

**Authors:** Yan Wang, Mengqi Wang, Hui Wang, Zhilin Dun, Lianwei Ren

**Affiliations:** 1 School of Energy and Environment Engineering, Zhongyuan University of Technology, Zhengzhou 450007, China; 2 School of Civil Engineering, Xi’an University of Architecture and Technology, Xi’an 710055, China; 3 School of Civil Engineering, Henan Polytechnic University, Jiaozuo 454000, Chinarenhpu@163.com (L.R.)

**Keywords:** experimental research, foundation reinforcement, waste concrete powder, waste brick powder, construction waste

## Abstract

Combined with a strategic policy for the recycling of construction waste, this paper puts forward a programme for treating a goaf foundation with waste concrete powder, waste brick powder, and cement grout (BCP cement grout). The BCP cement grout is prepared by replacing part of the waste concrete powder with waste brick powder, and the changeable rule of the setting time, water separation rate, stone rate, and viscosity of the grout with different rates for replacement are studied. X-ray diffraction (XRD) and scanning electron microscopy (SEM) are carried out. The mineral composition is analyzed and the microscopic mechanism is studied. The results show that the ratio of BCP cement grout developed by the experiment is reasonable; the unit cost is less than 160 yuan/t, which is 40% lower than that of pure cement grout; and both a good economic effect and good environmental effect are obtained. It has the advantages of low water separation rate, high stone rate, and low viscosity. When the ratio of cement powder: waste concrete powder: waste brick powder is 3:5:2 or 3:6:1, the 28 d compressive strength of the stone body is more than 2 MPa, meeting the filling requirements for a goaf foundation. It is not only technically feasible but also economically reasonable to apply construction waste powder to fill the goaf foundation. Recycling and utilizing construction waste can also achieve the ecological restoration of mined-out areas, highlighting the ecological benefits.

## 1. Introduction

In China, a contradiction is becoming increasingly prominent, that is, the increase of old coal mining areas and the shortage of land resources. Nowadays, in order to save land resources, some important infrastructure has to been built on the complex strata with bad conditions such as mined-out areas and fracture zones. This may cause changes in the original stress state of the foundation soil due to the disturbance of new building loads. Therefore, in order to carry out engineering activities on the goaf site, it is necessary to conduct research on the investigation and handling of the goaf foundation. It has been proven that filling reinforcement is one of the most commonly used methods for mined-out areas [1]. However, the traditional cement slurry [2,3] seriously restricts the application and development of the technology because of its disadvantages such as high water separation rate, low stone rate, large dispersion, serious waste of resources, high treatment cost, etc. Therefore, the treatment technology of mining areas is becoming an important research field [4,5].

On the other hand, the construction waste produced by demolition in China is up to billions of tons every year, mainly including waste concrete and waste clay bricks, which not only occupies land resources, but also pollutes the environment [6]. How to recycle construction waste scientifically, reasonably, and efficiently at home and abroad has become a research topic. Because the reinforcement mechanism of grouting in mined-out areas is mainly based on filling and compaction [7], and the requirements for material strength are not high, many experts and scholars at home and abroad are committed to the research on the recycling of construction waste and have made certain achievements in using construction waste for filling mined areas. In this study, a new treatment method for the grouting and filling of a goaf foundation was proposed. This not only enhances the top connection effect of grouting and filling, but also improves the utilization rate of construction waste, further reducing filling costs, and has important research value.

## 2. State of the Art

In terms of grouting materials, Portland Cement Clinker has the advantages of good flowability, adjustable setting time, and strong adaptability, so it is widely used [8,9,10]. However, its disadvantages during the preparation process—for example, long setting time, high cost, and high energy consumption—do not meet the requirements of green ecological engineering construction [11,12,13]. With the proposal of the concept of green and sustainable development, a large number of researchers have begun to develop solid waste into grouting materials to achieve environmental protection and low-cost construction. Kwon et al. [14,15] studied the relationship among the setting time, fluidity, viscosity, and stone rate of grout under different water–cement ratios, solid-phase ratios, and other conditions for fly ash–cement grouting materials. Perez et al. [16,17,18,19] developed solid waste as a new grouting material to achieve the purpose of recycling, energy saving, and material saving.

In terms of the recycling of waste concrete, scholars have begun to try to use recycled aggregate in fields with low requirements for mechanical properties. Sanchez et al. [20] prepared hot mix asphalt mixture (HMA) from natural coarse aggregate (NCA). The results showed that the two materials had similar characteristics, but HMA was more environmentally friendly; Zhou et al. [21] used the recycled aggregate of construction waste as an inorganic mixture, and the results showed that the strength was significantly improved; Kim et al. [22] used waste concrete powder (WCP) instead of silica fume to produce extruded concrete panels; and Zheng et al. [23,24,25] recycled the waste concrete powder and achieved good results. The crushing and processing technology of waste concrete in foreign countries includes many processing equipment and has high production costs. However, the crushing and processing technology in China, combined with the basic national conditions of China, mostly chooses manual methods for the preliminary sorting of waste concrete blocks. Further research should be conducted in terms of the selection of the production equipment and aggregate recycling rate.

In terms of the recycling of waste clay bricks, the research mainly focuses on using waste clay brick powder as an aggregate for building mortar or concrete. Cao et al. [26,27] crushed the waste bricks into recycled brick aggregates, and carried out mechanical property tests. The results showed that recycled brick aggregates had better thermal insulation and fire resistance properties; Ortega et al. [28,29,30] used waste brick powder to replace part of the clinker to produce recycled cement, and carried out a microstructure analysis. It was found that the performance was even better than ordinary Portland cement. Tang et al. [31,32] have studied the effect of partially replacing cement with waste clay brick powder on the performance of cement mortar. The results show that clay brick powder can reduce the early compressive strength of recycled mortar, but the later compressive strength is greater than that of ordinary mortar.

In terms of the design and performance of the concrete composite mix, Huang et al. [33] investigated the impact of different mix proportions on the performance of geopolymer concrete, and conducted experimental studies on the effects of the ratio of fly ash to granulated blast furnace slag, modulus, and content of sodium silicate on the macro-mechanical properties; Chen et al. [34] studied the performance of recycled concrete with different amounts of clay brick aggregates and found that the maximum of apparent density can be reduced by 17%, but the maximum of compressive strength can be reduced by 40%. The study by Cachim et al. [35] showed that, when the replacement rate of clay bricks was not high, the loss of the concrete mechanical energy was lower. However, when the replacement rate was higher than 30%, its various properties would be significantly reduced. Yang et al. [36] used waste clay bricks to completely replace natural sand and stones to prepare recycled concrete, and investigated the effects of the substitution rate of the water–cement ratio, cement dosage, fly ash, and silica fume on the performance of recycled concrete. Currently, the research on concrete mixed with waste clay brick aggregates mainly focuses on the impact of replacing natural aggregates on performance, with less research on the impact of mixing parameters on performance after completely replacing the natural aggregates.

Recycled concrete is mainly used in fields with low requirements for mechanical properties. For abandoned clay bricks, recycled aggregates, recycled sand, and recycled cement used by them have not been widely used due to the disadvantages such as low strength, high water absorption, and poor quality. Moreover, in practical applications, it is difficult to obtain the optimal mixing ratio through traditional proportioning methods because a few multiple variables such as the economy, environmental protection, and energy consumption need to be considered. Overall, the recycling and utilization of construction waste in China is still in a primary state with disorderly management, simple technology, and extremely low recycling efficiency.

With the increasing awareness of environmental protection, a large amount of solid waste has been applied to the field of grouting reinforcement. However, after years of development, the grouting and filling materials for mining areas are still mainly cement grout and cement fly ash grout. In order to develop more grouting materials for environmental protection which are suitable for mining areas, the research group will use waste concrete and waste clay bricks, which are widely sourced and economical as consolidation materials, replacing some cement, to prepare waste brick powder, waste concrete powder, and cement grout (BCP cement grout for short). The basic properties of BCP cement grout were thoroughly and systematically tested from both the macro and micro perspectives. The research results have important theoretical significance in promoting the recycling and utilization of construction waste, protecting the environment, and saving energy.

## 3. Experimental Materials and Schedules

### 3.1. Raw Materials

The cement used in the experiment was the sturdy brand P · O_42.5_ grade ordinary Portland cement produced in Jiaozuo City, China, and its main physical and mechanical properties are shown in Table 1.

**Figure 1:**
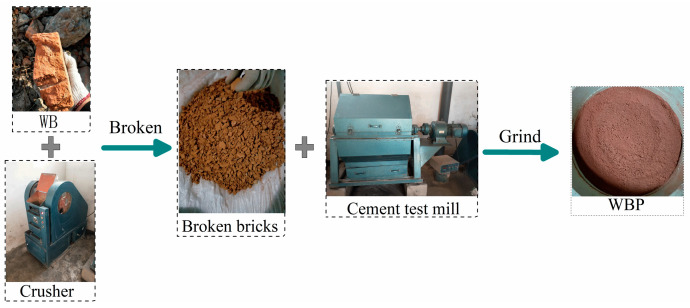
Processing steps of waste clay bricks.

**Figure 2:**
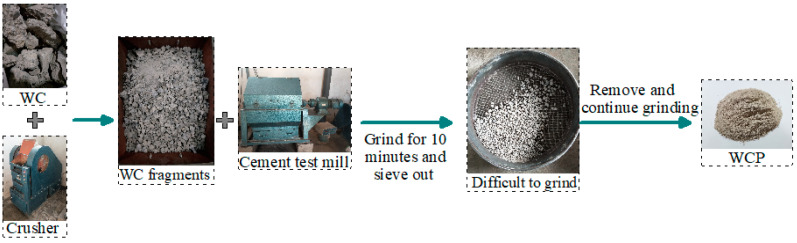
Processing steps of waste concrete.

The waste concrete and clay bricks used in the test were taken from the construction waste produced from the demolition of old buildings in Henan Polytechnic University. Figure 1 and Figure 2 show the processing steps of waste clay bricks and waste concrete. For waste concrete, it can be seen that there is one more screening step than waste clay bricks when processing, which is due to its high hardness, uneven particles, and different abrasion resistance for different particles.

**Figure 3:**
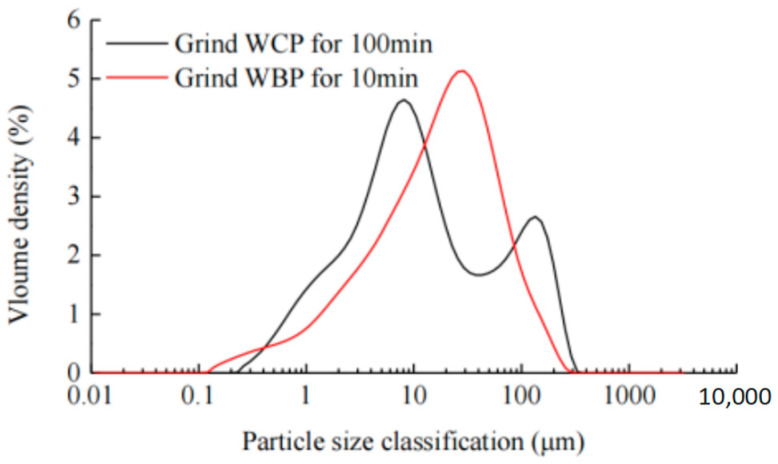
Particle size grading of two kinds of powders. Reproduced with permission [37,38], 2022, Springer and Hindawi.

The particle sizes of waste brick powder and waste concrete powder were analyzed with a laser particle size analyzer, and the particle size distribution curves of the two powders at different grinding times were obtained. Figure 3 showed two particle size distribution curves: one is from the waste concrete powder for grinding 100 min, the other is from the waste brick powder for grinding 10 min. It can be seen that the particle size distribution of brick powder is uniform; however, the waste concrete powder has a strong non-uniformity and high proportion of coarse particles.

**Figure 4:**
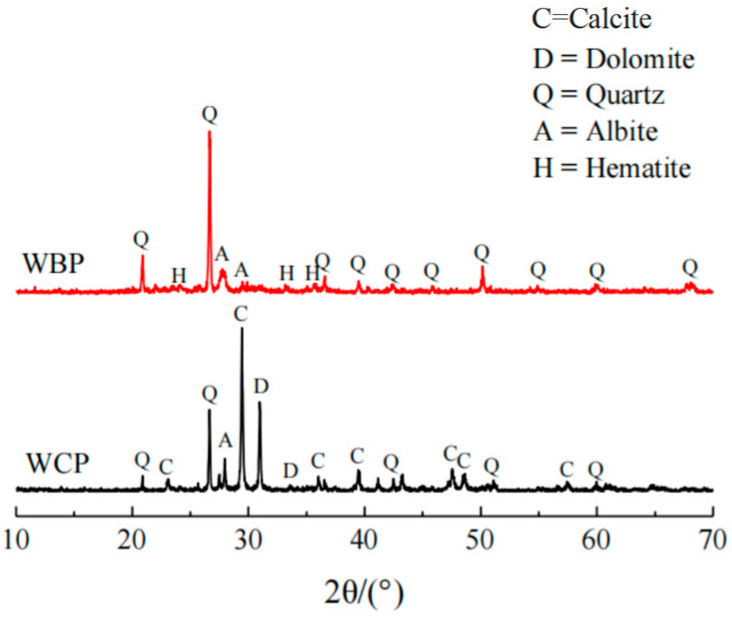
XRD Spectrum of Powder. Reproduced with permission [37,38], 2022, Springer and Hindawi.

**Figure 5:**
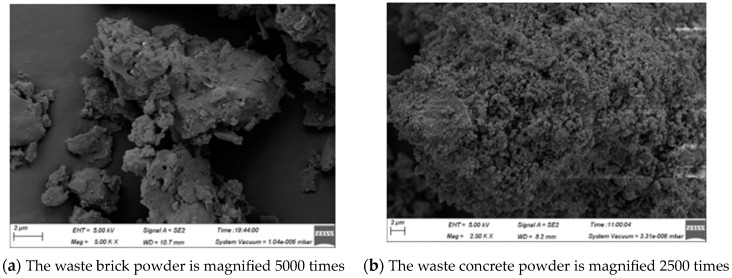
SEM of powder.

The basic composition of waste brick powder and waste concrete powder is obtained by X-ray diffraction (XRD) test (Figure 4). It can be seen that the waste brick powder is mainly composed of quartz, hematite, and albite calcian, in which SiO_2_ is beneficial for improving the strength of concrete and has strong corrosion resistance. There is dolomite in the waste concrete powder compared to the waste brick powder, in which CaCO_3_ is not conducive to the strength of concrete system. A scanning electron microscope (SEM, Merlin Compact field emission scanning electron microscopy, Germany) was used to analyze the internal microstructure of the two powders, and the SEM diagram (Figure 5) was obtained. It is apparent that the structure of waste brick powder is relatively dense, and the structure of waste concrete powder is relatively loose, and both surfaces are not smooth. From the diagram of XRD Spectrum of Powder, it can be seen that the ground brick powder has a certain gradation and a relatively uniform particle distribution, which is the reason for the low water absorption of the brick powder and the high water precipitation rate of the slurry. The structure of waste concrete powder is relatively loose and its surface is not smooth. It contains particles wrapped and bonded by gel, and a large number of pores, indicating that the surface of waste concrete powder is porous and has strong water absorption, so the slurry mixed with waste concrete powder has strong water absorption and high viscosity.

In accordance with the requirements of <Method of testing cements- Determination of strength (GB/T 17671-1999) [39]>, the two kinds of powders are mixed, stirred, and cured with cement, standard sand, and water, respectively. Through testing and calculation, the activity index of waste brick powder is 66%, and that of waste concrete powder is 57.4%. It can be seen that brick powder not only meets the requirements of fineness, but also has potential pozzolanic activity. The activity of waste concrete powder is relatively low, although the fineness meets the requirements.

To clarify the chemical composition of the two powders and cement, they were tested using a Zetium X-ray fluorescence diffractometer from Panaco, The Netherlands. The test results are shown in Table 2.

### 3.2. Test Scheme Design of BCP Cement Grout

#### 3.2.1. Test Purpose

According to literature [37,38], waste brick powder has the advantages of short grinding time, less energy consumption, and high pozzolanic activity, but the disadvantages of poor water absorption, high water separation rate, and low stone rate. The waste concrete powder has the advantages of strong water absorption, low water separation rate, and high stone rate, but it has the disadvantages of long grinding time, high energy consumption, high viscosity, and poor fluidity. Based on the concept of complementary advantages, the two kinds of powders are mixed together, and it is expected that all performance indicators of the mixed grout can meet the requirements of grouting and filling of goaf foundation without adding any admixture.

#### 3.2.2. Test Contents and Methods

The test content includes macroscopic performance testing and microscopic performance testing. According to the mechanism of filling and reinforcement of goaf foundation, macroscopic performance testing mainly includes viscosity, setting time, water precipitation rate and stone rate, and compressive strength. Micro performance testing mainly includes XRD and SEM.

(1) Viscosity

**Figure 6:**
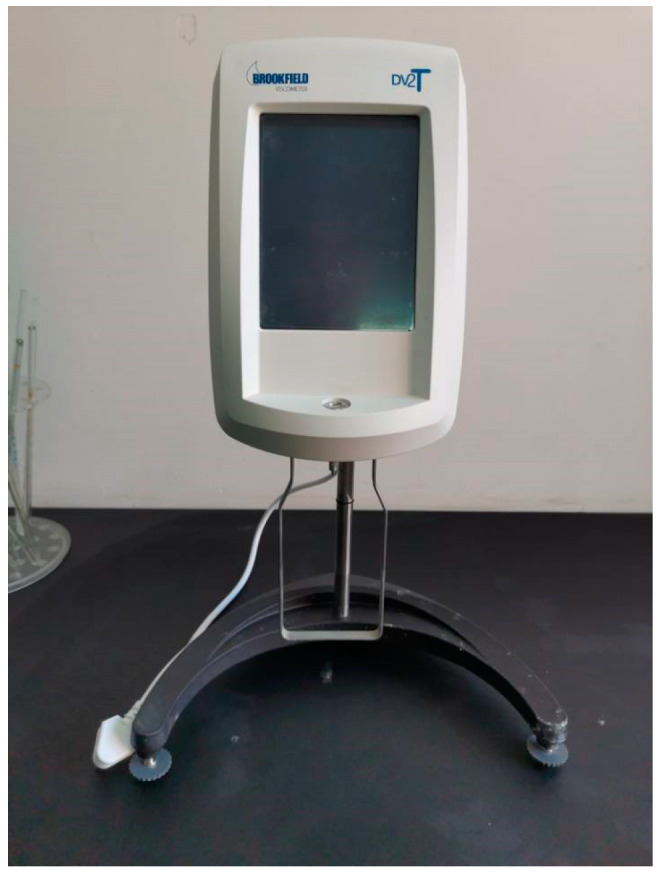
Brookfield DV2T viscometer.

The viscosity characterizes the fluidity of the slurry, and the smaller the viscosity, the better the fluidity. The viscosity test was conducted by using the Brookfield DV2T viscometer from the United States, as shown in the Figure 6.

(2) Setting time

**Figure 7:**
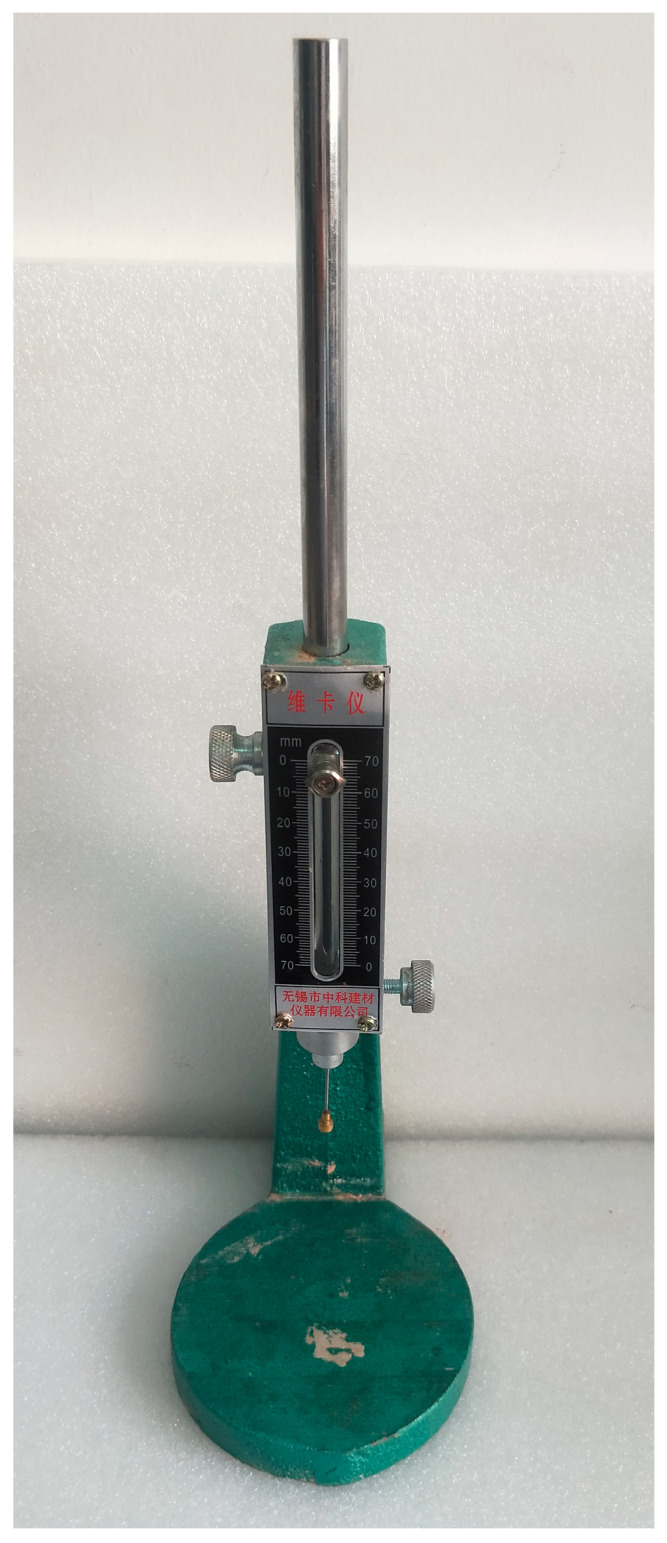
Vicat apparatus.

The setting time is divided into initial setting time and final setting time. The initial setting time should not be too short, and the final setting time should not be too long. The setting time shall be tested by using the Vicat apparatus in accordance with <Test methods for water requirement of normal consistency, setting time and soundness of the portland cement> (GB/T 1346-2011) [40], as shown in the Figure 7.

(3) Water precipitation rate

The water precipitation rate of the slurry refers to the rate at which the slurry releases water due to the sedimentation of particles in a stationary state. It is an important indicator with which to measure the stability of the slurry. The higher the water precipitation rate is, the more unstable the slurry is considered, which will seriously affect the grouting effect. In this experiment, a measuring cylinder was used to test the water precipitation rate of the slurry.

(4) Stone rate

The stone rate of slurry refers to the ratio of the volume of the slurry after solidification to the original volume of the slurry. The slurry with high stone rate after solidification can effectively fill voids, holes, and cracks in the goaf foundation. In order to achieve better filling effect of the goaf foundation, it is usually required that the stone rate of the slurry be not less than 80%. The measurement of stone rate is still tested by using a measuring cylinder.

(5) Compressive strength

The grouting and filling of the goaf foundation does not require high strength of the slurry. According to the <Technical code for ground treatment of buildings in coal mine goaf (GB 51180-2016) [41]>, during the grouting design stage, the uniaxial compressive strength of indoor curing test blocks should not be less than 2 MPa. Test block was cast in triplet test mould, whose side length was 70.7 mm.

XRD and SEM for microscopic performance testing have been given a detailed introduction in Section 3.1, which will not be provided here.

#### 3.2.3. Test Scheme Design

According to <Technical code for ground treatment of building in coal mine goaf (GB 51180-2016)>, the water–solid ratio of grouting filling slurry for goaf foundation is generally 1:1–1:1.3. The fixed water–solid ratio in this test is 1:1.1, and pure cement slurry (water–cement ratio is 1:1.1) is set as the control group.

When designing test scheme of BCP cement grout, waste concrete powder–cement grout (WCP cement grout for short) is prepared first, and then part of the waste concrete powder is replaced with waste brick powder while maintaining the total mass and cement powder mass unchanged to study the change rules of various performance indicators of grout under different replacement rates. In order to improve the utilization rate of construction waste powder as much as possible, this test selects three groups of 70% (WCP70), 80% (WCP80), and 90% (WCP90) of concrete powder for carrying out subsequent study of proportion tests. The detailed test scheme is shown in Table 3.

## 4. Results and Discussion

### 4.1. Characteristic Analysis of BCP Cement Grout

#### 4.1.1. Viscosity

**Figure 8:**
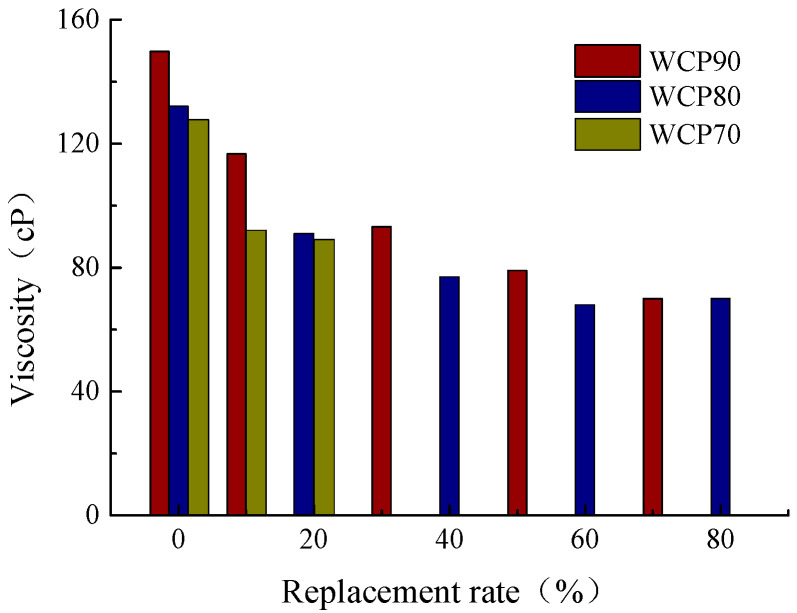
Viscosity at different substitution rates.

The variation law of slurry viscosity for WCP70, WCP80, and WCP90 under different replacement rates is shown in Figure 8. It can be seen from the figure that the viscosity of grout tends to decline after some waste concrete powder is replaced by waste brick powder, indicating that the addition of waste brick powder can effectively reduce the viscosity of the slurry and improve the groutability of the slurry. With the increase in replacement rate, the decrease in slurry viscosity decreases, which indicates that the influence of waste brick powder on viscosity decreases gradually. For the WCP70 group, due to the lower original viscosity of the slurry, when the substitution rate is more than 10%, the slurry has good fluidity and groutability. For the WCP80 group, when the replacement rate is more than 20%, it has better liquidity and groutability. For the WCP90 group, it has the highest slurry viscosity; when the replacement rate is equal to or more than 50%, the slurry has better fluidity and groutability.

**Figure 9:**
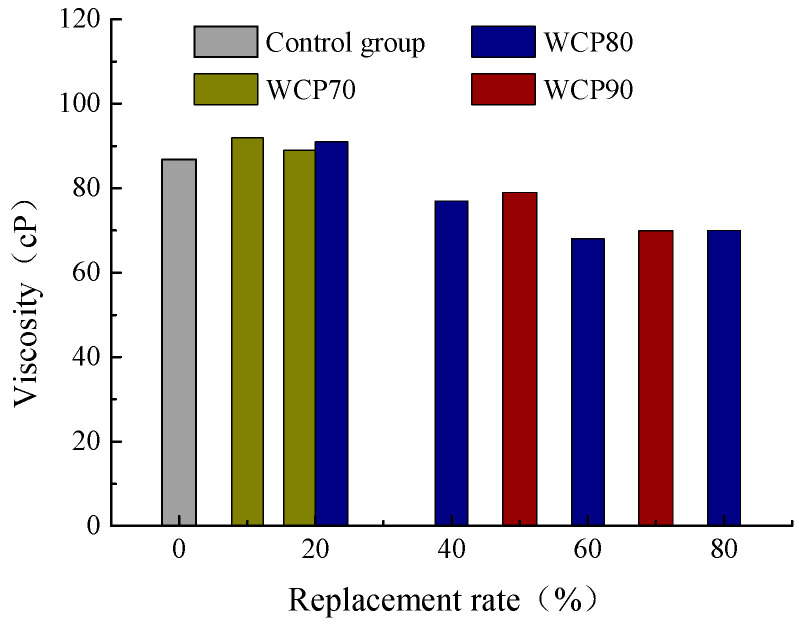
Comparison of viscosity after adding suitable waste brick powder.

Three groups are compared and analyzed with the control group (pure cement slurry), and the results are shown in Figure 9. It can be seen that the slurry viscosity of the WCP70 group is slightly higher than that of the control group, and it is considered to have a similar viscosity to the control group. The WCP50WBP20 group with a replacement rate of 20% is preferred as the slurry for the grouting treatment of the goaf foundation. For the WCP80 group, when the replacement rate is 20%, the viscosity is higher than the control group. When the replacement rate is equal to or more than 40%, the viscosity is lower than the control group, with better mobility. For the WCP90 group, when the replacement rate is equal to or more than 50%, the viscosity of the slurry is lower than that of the control group, and it has good fluidity. In addition, when the replacement rate is 20%, the slurry viscosity of the WCP70 group is lower than that of WCP80 group. This is because the content of waste concrete powder in the slurry of the WCP80 group is 10% higher than that of the WCP70 group; therefore, the component with the higher waste concrete content is more viscous. Comparing the WCP40WBP40 group with the WCP40WBP50 group, it is found that the viscosity of the WCP40WBP50 group is slightly higher than that of the WCP40WBP40 group, indicating that the waste brick powder has a slightly greater effect on the viscosity of the slurry than the cement.

#### 4.1.2. Water Separation Rate and Stone Rate

**Figure 10:**
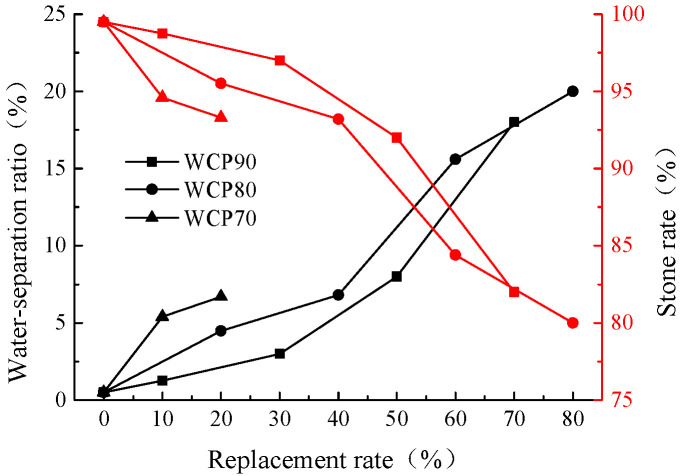
Water separation rate and stone rate under different replacement rates.

From the previous test results (References [37,38]), it can be seen that the change of viscosity has a great influence on the water separation rate and stone rate of the slurry. The water separation rate and stone rate of the slurry under different replacement rates are shown in Figure 10. From the figure, we found that, with the increase of the content of waste brick powder, the water separation rate of the slurry of each group is increasing and the stone rate is decreasing, which indicates that there is a certain influence on the filling effect of the slurry. In addition, the sum of the water separation rate and the stone rate of the slurry is still equal to 1, indicating that the slurry with a composite ratio of these two powders will not display obvious expansion and contraction during the curing process. In addition, it should be noted that, for the slurry of the WCP90 group, although there is good fluidity after the replacement rate exceeds 50%, the water separation rate of the slurry increases rapidly and the stone rate decreases rapidly, which seriously affects the grouting filling effect of the goaf foundation. Therefore, the replacement rate should not exceed 50%. For the slurry of the WCP80 group, when the replacement rate exceeds 40%, the water separation rate also increases rapidly and the stone rate decreases rapidly, so the replacement rate should not exceed 40%.

**Figure 11:**
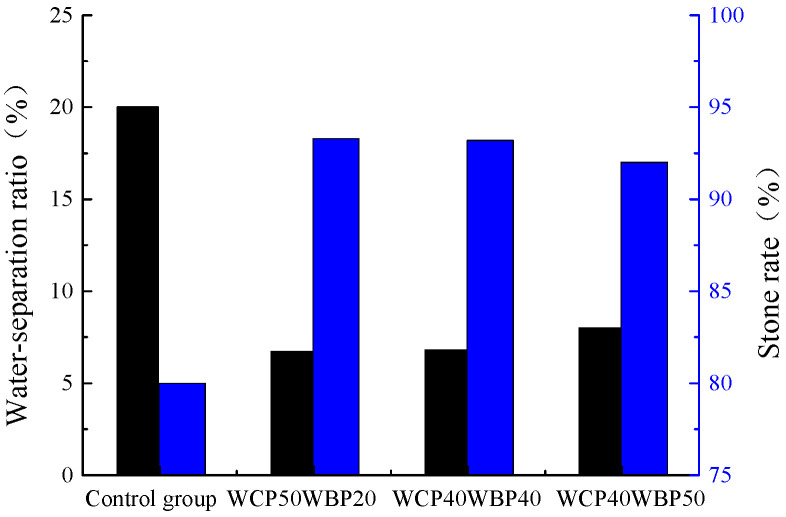
Water separation rate and stone rate of suitable components.

Combining the analysis results of Section 4.1.1 and Section 4.1.2, the slurries of the WCP50WBP20 group, WCP40WBP40 group, and WCP40WBP50 group which meet the requirements of viscosity, water separation rate, and stone rate are compared with the control group, and the results are shown in Figure 11. It can be seen from the figure that the water separation rate and stone rate of the newly prepared three groups are much higher than the control group, and the stone rates of the slurry are all above 90%. When applied to the foundation grouting of the goaf, it will show a good filling effect. By comparing the three newly prepared slurries alone, it is found that, although the stone rate of the WCP40WBP50 group is above 90%, the water separation rate is higher and the stone rate is lower than the other two groups. This is because the content of waste concrete powder with strong water absorption in the slurry of the WCP40WBP50 group is not high, and the content of waste brick powder is the highest among the three groups. The stone rate of the slurry will be reduced and the water separation rate will be increased with the increase in the content of waste brick powder. Therefore, the slurry of the WCP40WBP50 group is slightly worse than that of the other two groups. Comparing the slurries of the WCP50WBP20 group and WCP40WBP40 group separately, it is found that there is no significant difference in the water separation rate and stone rate between the two groups, but the content of waste concrete powder in the slurry of the WCP50WBP20 group is 10% higher than that of the WCP40WBP40 group. This phenomenon may be due to the fact that the cement content in the slurry of the WCP50WBP20 group is 30%, while the water precipitation rate of the slurry of the WCP70 group is higher than that of the WCP80 group, and the content of waste brick powder in the slurry of the WCP40WBP40 group is 20% higher than that of the WCP50WBP20 group, and the water absorption of waste brick powder is lower than that of waste concrete powder, but higher than that of cement particles. Therefore, the water separation rate and stone rate of slurry of the WCP40WBP40 group can reach the same level as that of the WCP50WBP20 group.

#### 4.1.3. Setting Time

**Figure 12:**
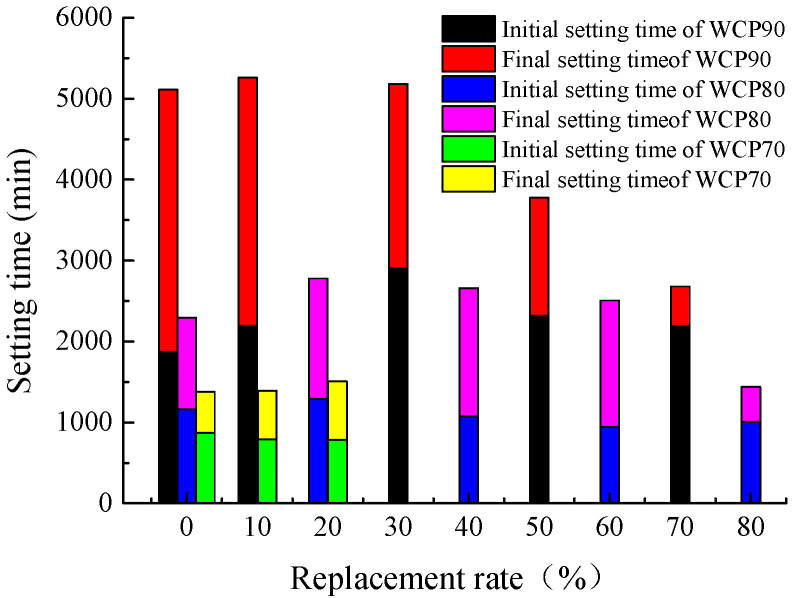
Setting time under different replacement rates.

The change rule of the setting time of the slurry after replacing some waste concrete powder with brick powder is shown in Figure 12. From the figure, it can be seen that the initial setting time and final setting time of the slurry in the WCP80 and WCP90 groups show a trend of increasing first and then decreasing, but a turning point occurs at 20%. When the replacement rate is equal to or less than 20%, the setting time increases with the increase in replacement rate, but when the replacement rate is equal to or more than 20%, the setting time begins to decrease with the increase in replacement rate, and the decrease is obvious. This is because, after replacing some waste concrete powder with waste brick powder, the setting time of the slurry will be prolonged to a certain extent. However, when the proportion of brick powder is too high, the water separation rate of the slurry is too large. At this time, a large amount of water is precipitated, and the water participating in condensation is reduced, which is equivalent to reducing the water–solid ratio of the slurry, so the setting time of the slurry is reduced. Therefore, when the replacement rate is equal to or less than 20%, the main factor affecting the setting time is the replacement rate; when the replacement rate is equal to or more than 20%, the main factor affecting the setting time is the slurry separation rate. For the WCP70 group, because only the replacement rate is set to 20% when the viscosity is optimized, the setting time does not decrease, but the development trend of the upward stage is consistent with the first two groups, it can be inferred that the change rule of the setting time is similar to that of the other two groups. In addition, comparing the initial setting time and final setting time of the slurry, it is not difficult to find that, after replacing the waste concrete powder with brick powder, the interval between the initial setting time and the final setting time of the slurry decreases, and the higher the replacement rate, the shorter the interval. This indicates that with the increase in the proportion of brick powder, the process from losing plasticity to producing strength is shortened.

**Figure 13:**
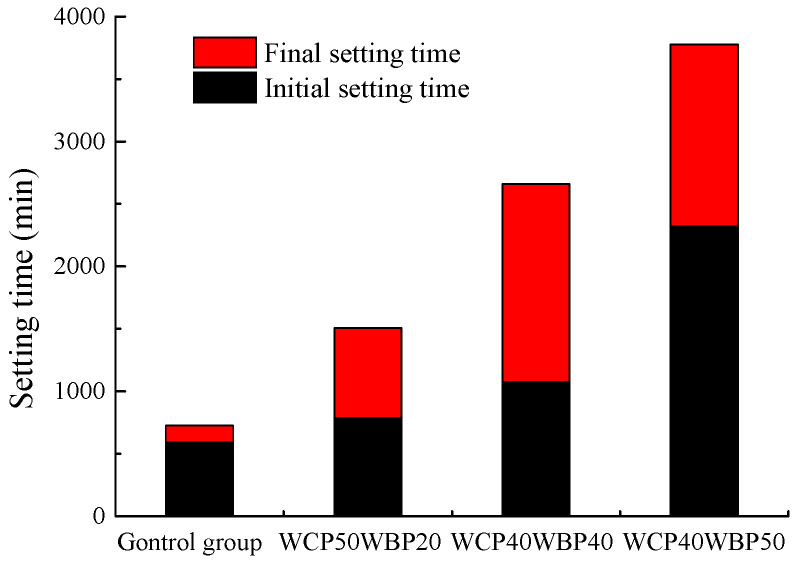
Setting time of suitable components.

Based on the analysis results of Section 4.1.1 and Section 4.1.2, the setting time of slurry for the WCP50WBP20 group, WCP40WBP40 group, and WCP40WBP50 group which meet the requirements of viscosity, water separation rate, and stone rate in the optimization process is compared with that of the control group. The results are shown in Figure 13. From the figure, it can be seen that the setting time of the slurry from short to long is the control group, WCP50 WBP20 group, WCP40 WBP40 group, and WCP40 WBP50 group. The setting time is quite different, indicating that the setting time of the slurry is mainly determined by the cement content: the higher the cement content, the shorter the setting time; while the lower the cement content, the longer the setting time. In addition, the setting time of the newly prepared three groups of slurry is longer than that of the control group, which is more conducive to the flow and diffusion of the slurry, but the setting time of the slurry of the WCP40WBP50 group is too long, which may affect the progress of the project activities, and should be carefully selected according to the site conditions.

#### 4.1.4. Compressive Strength

**Figure 14:**
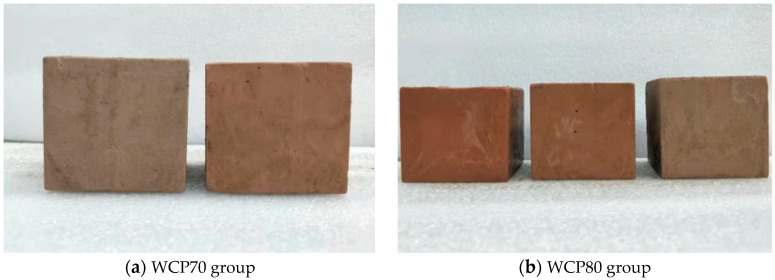
Specimen after demoulding.

The slurry with different replacement rates is poured into a triple test mold with a side length of 70.7 mm. After curing for 48 h, the mold is removed, and the demoulded specimens are continuously cured for 3 days, 7 days, and 28 days, for the uniaxial compressive strength test. Figure 14 shows two groups of specimens after demoulding, of which Figure 14a is the specimen of the WCP70 group with different replacement rates. The specimens in the figure are the WCP60WBP10 group and WCP50WBP20 group from left to right. From the figure, we can see that the WCP50WBP20 group with a higher brick powder content is shorter than the WCP60WBP10 group with a lower brick powder content, indicating that the higher the brick powder content, the lower the stone rate of the slurry. In addition, it is not difficult to see from the figure that the color of the WCP50WBP20 group is slightly deeper than that of the WCP60WBP10 group. This is because the brick powder content of the WCP50WBP20 group is higher, making the color of the specimen red. Figure 14b shows the specimens of the WCP80 group poured at different replacement rates. The specimens in the figure are the WCP20WBP60 group, WCP40WBP40 group, and WCP60WBP20 group from left to right. It can be found from the figure that, with the decrease in brick powder content, the height of the specimens is getting higher and higher. It is, once again, confirmed that the higher the replacement rate, the lower the slurry stone rate. Not only that, because the brick powder content gradually decreases, the color of the specimens in this group becomes lighter from left to right.

**Figure 15:**
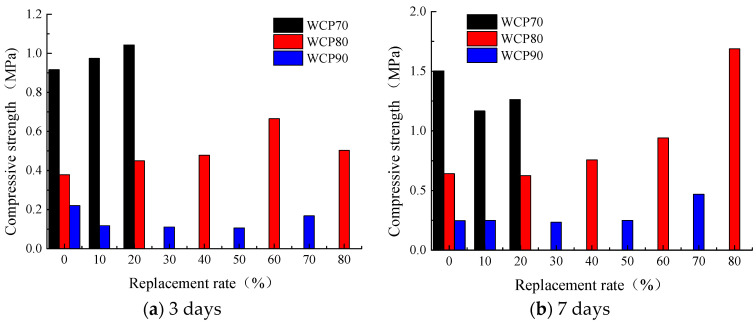

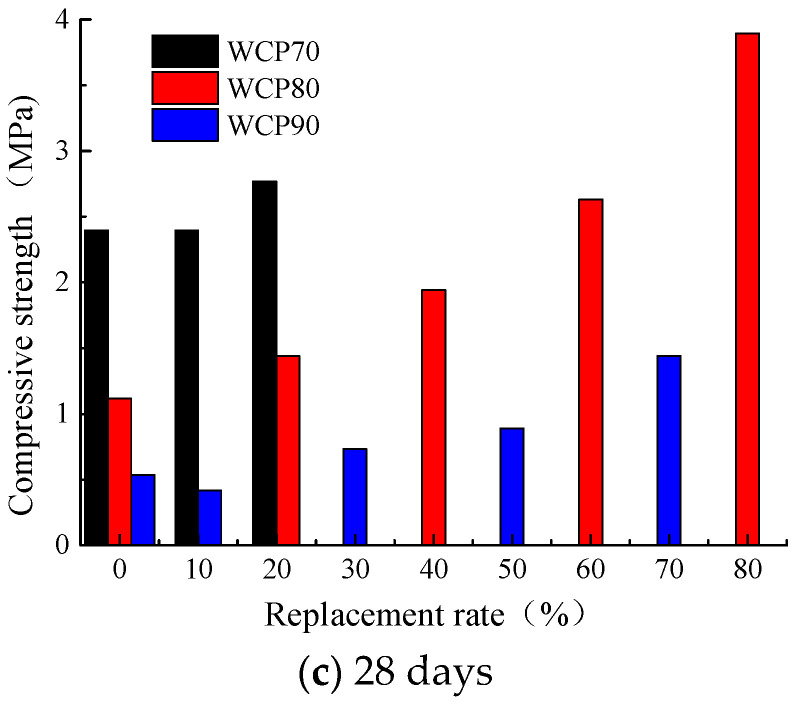
Compressive strength of different ages under different replacement rates.

The compressive strength of the cured test blocks under different replacement rates is shown in Figure 15. It can be seen from the figure that, with the increase in the proportion of brick powder, the 3-day compressive strength of the WCP70 group specimens increases, and the 7-day compressive strength and 28-day compressive strength show a trend of decreasing first and then increasing, indicating that the strength of the specimens after adding brick powder increases slightly in the early and middle stages, and the strength growth is large in the middle and late stages. With the increase in replacement rate, the 3-day compressive strength of the WCP80 group specimens show an overall upward trend. When the replacement rate is 60%, a singularity appears, which may be caused by experimental errors, and the 7-day compressive strength and 28-day compressive strength continue to increase. With the increase in replacement rate, the compressive strength of the WCP90 specimens at each age decreases slightly and then increases, which shows that, when the replacement rate is small, the compressive strength of the specimens is not stable. When the replacement rate is large, the water separation rate of the slurry and the pozzolanic activity of waste brick powder can significantly improve the later strength of the specimens.

**Figure 16:**
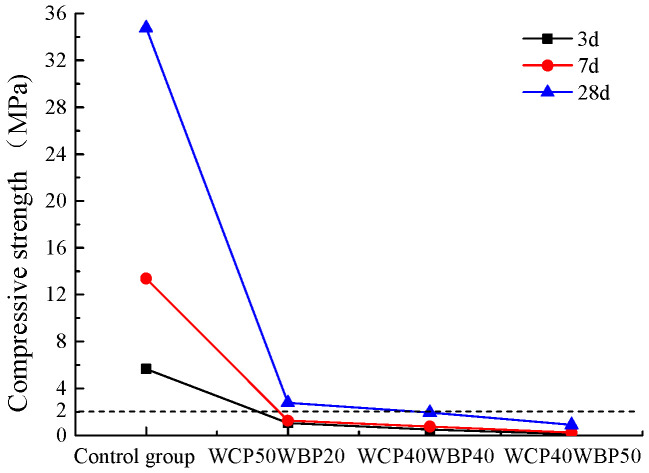
Compressive strength of suitable components.

Combined with the analysis results of Section 4.1.1 and Section 4.1.2, the compressive strength of the WCP50WBP20 group, WCP40WBP40 group, and WCP40WBP50 group that meet the requirements of viscosity, water separation rate, and stone rate in the optimization process was compared with the control group; the comparison results are shown in Figure 16. It can be seen from the diagram that the compressive strength of the newly prepared three groups of slurry at each age is much lower than that of the control group; this is because the cement content in the newly prepared slurry is only 10~30%, which is much lower than the cement content in the pure cement slurry, so its compressive strength is low. The grouting treatment of the goaf foundation has low requirements on the strength of the stone body of the slurry. The specification requires that the indoor curing strength of the specimen in the design stage be not less than 2 MPa. From the figure, it can be found that the 28 d compressive strength of the WCP50WBP20 group is greater than 2 MPa, which is suitable for the filling treatment of the goaf foundation. In addition, the 28 d compressive strength of the WCP40WBP40 group of specimens is close to 2 MPa, which can be used for voids and fissures in the goaf foundation according to the site conditions.

### 4.2. Micro Mechanism of BCP Cement Grout

#### 4.2.1. XRD

**Figure 17:**
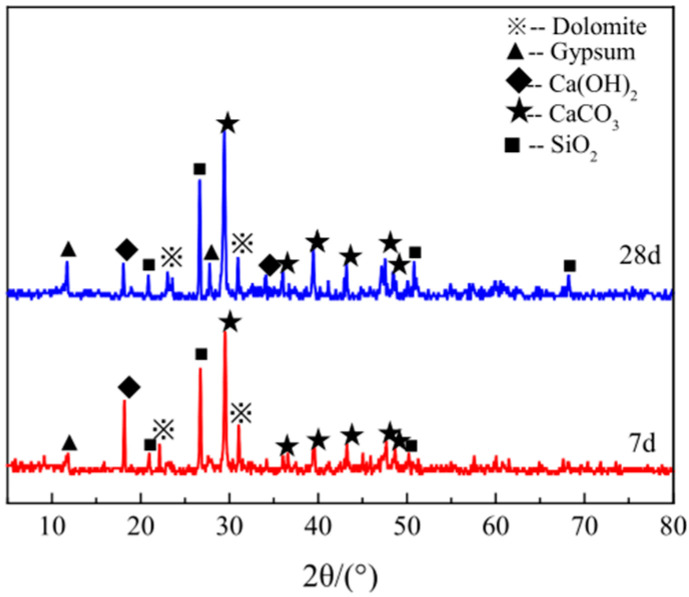
XRD spectra of WCP50WBP20 group at different curing periods.

Take samples of the WCP50WBP20 group of specimens with a 7 d and 28 d curing period and grind them into powder for the XRD analysis. The results are shown in Figure 17. The diffraction peaks of CaCO_3_ and SiO_2_ in the sample are stronger than those of other crystals, and there are more diffraction peaks. This is because the content of CaCO_3_ and the degree of crystallization in waste concrete powder are higher, and the main component of fine aggregate sand is quartz, so the diffraction peaks of SiO_2_ crystals in the sample are higher. By observing the spectra of different periods, it was found that the crystal types in the samples, which were cured for 7 d and 28 d, were basically the same, indicating that new crystals would not occur or the original crystals would not be lost in different curing periods. In addition, it can be seen from Figure 17 that, although the positions of the peaks are basically the same, the peak heights change to varying degrees, indicating that changes in the curing period have an impact on the crystallinity of the phase.

#### 4.2.2. SEM

**Figure 18:**
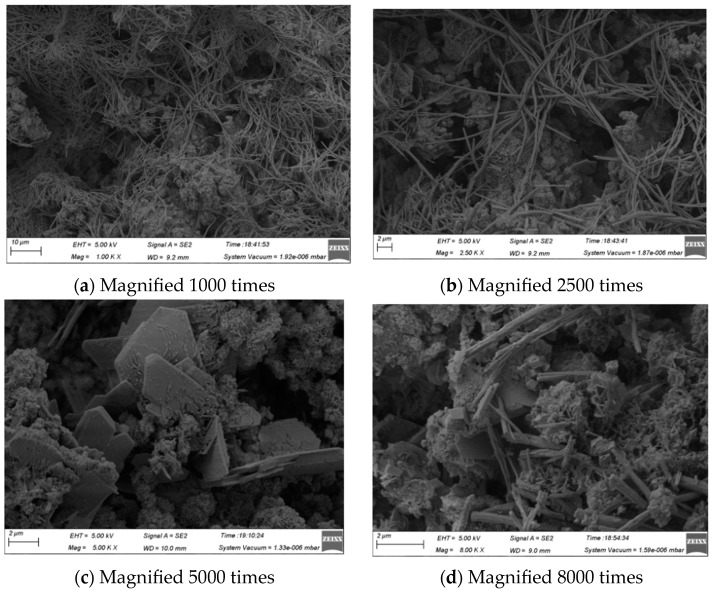
SEM diagram of WCP50WBP20 specimen after 7 days of curing.

Take a sample of WCP50WBP20 whose component ratio is 3:2:5 of cement, brick powder, and concrete powder for SEM microscopic scanning and analyze the microscopic morphology changes at 7 and 28 days. Figure 18 shows the morphology of the specimen after 7 days of curing. It can be seen from Figure 18a that the surface of the sample is covered with a lot of fibrous AFt and pores of various specifications, indicating that the early structure of the sample is relatively loose. It can be seen from Figure 18b that there are many gel and other substances under AFt, and AFt is mainly in a network structure, covering the surface of these substances. A large amount of sheet-like Ca(OH)_2_ can be seen from Figure 18c, which creates an alkaline environment for the later pozzolanic reaction of the sample. It can be seen from Figure 18d that, in addition to the above substances, there are cubic CaCO_3_ crystals, indicating that part of the construction waste powder plays an important role in filling and exerting its micro aggregate effect.

**Figure 19:**
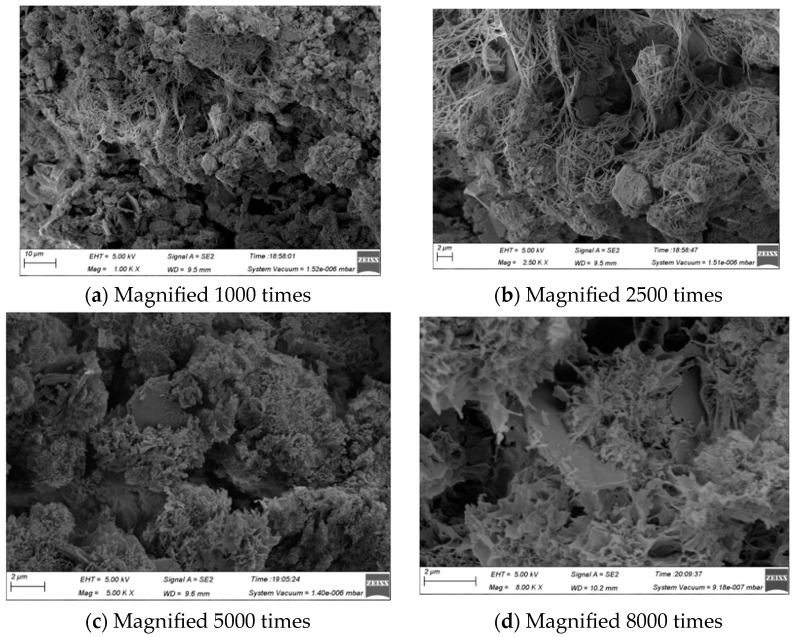
SEM diagram of WCP50WBP20 specimen after 28 days of curing.

Figure 19 shows the microscopic morphology of the specimen after 28 days of curing. Comparing Figure 19a with Figure 18a, it can be seen that the AFt distribution in the later period becomes dense, not as loose as in the earlier period, and the number of pores decreases, and the pore diameter becomes smaller. Comparing Figure 19b with Figure 18b, it is found that the AFt covering the surface of gel and other substances in the early stage is interspersed between these substances in the later stage, which indicates that a large amount of gel generated in the later stage will bond the originally loose AFt, thus making the AFt structure in the sample more compact. Comparing Figure 19c with Figure 18c, it is found that a large amount of Ca(OH)_2_ produced in the early stage becomes less in the later stage, which may be because part of Ca(OH)_2_ participates in the pozzolanic reaction in the later stage and is converted into CSH gel, and part of Ca(OH)_2_ is wrapped and bonded by gel. Comparing Figure 19d with Figure 18d, it is found that only CSH gel can be seen in the later stage; this is because a large number of the gel wraps and bonds the AFt, CaCO_3_, and other substances in the earlier stage to provide strength for the specimen.

## 5. Conclusions

The properties of waste brick powder–cement grout (WBP cement grout) and waste concrete powder–cement grout (WCP cement grout) were analyzed in [37,38], respectively. According to the research results in this paper, the performances of the three kinds of grout can be compared to find out their corresponding advantages, so that the most suitable grout proportion can be selected according to the requirements in practical engineering.

(1) Macroscopic performances: In terms of viscosity, the WCP cement slurry and BCP cement slurry are higher, while WBP cement slurry is the lowest and has the best fluidity. In terms of the water separation rate and stone rate, the water separation rate of the three groups is much lower than that of the control group, and the stone rate is much higher than that of the control group. Among them, the water separation rate of the WCP group is the lowest (about 5%), and the stone rate is the highest (up to 95%). In terms of setting time, the WBP group has higher initial setting time and final setting time. In terms of compressive strength, the 3-day compressive strength of the WBP group is much lower than that of the WCP group and BCP group, the 7-day compressive strength is slightly lower than that of the WCP group and BCP group, and the 28-day compressive strength is basically the same or even slightly higher than that of the WCP group and BCP group.
(2) Microscopic performances: The XRD analysis shows that there are few crystal phases in the WBP group. As the main raw material of this group is brick powder, the number of diffraction peaks of SiO_2_ is the largest, and the peak strength is the largest. The crystal phase types in the WCP group and BCP group are basically the same, but the diffraction peaks of CaCO_3_ crystal in the WCP group are the strongest and the number of diffraction peaks is the largest. The SEM analysis shows that the AFt generated from the hydration of samples in the WCP group and WBP group is in the form of short columns or short needles interspersed between gel, while the AFt of samples in the BCP group is in the form of large radial fibers, indicating that the composite action of the two kinds of powders will affect the microscopic morphology of ettringite to some extent.
(3) Economic and environmental performances: Through calculation, the unit cost of pure cement slurry is 261.91 yuan/t, and the three kinds of slurries have significant economic and environmental characteristics compared with pure cement slurry. Due to the long grinding time of waste concrete powder, WCP cement grout is the most expensive of the three kinds of grout, at 182.55 yuan/t, but the stone rate is the highest. WBP cement slurry is the cheapest, at 92.77 yuan/t, with the best economic and environmental performances. BCP cement slurry has a medium economic performance, at 154.83 yuan/t, but it can fully utilize the performance advantages of waste concrete powder and waste brick powder, and no other additives need to be consumed during application.
(4) Limitations: This experiment mainly studied the basic properties of the slurry and the compressive strength of the stone body. However, the internal environment of the mined-out areas is complex, and the buildings have a service life of 50–70 years. Therefore, it is necessary to conduct further research on the durability of the slurry material in the goaf field.
(5) Prospects: Indoor model tests should also be conducted to study the diffusion radius of grout with different ratios in different mined-out areas. The mechanical properties of the stone body in the model test should be tested by taking the cores and the relationship between the compressive strength of the stone body after grouting, and the compressive strength of the indoor pouring specimen should be studied.
(6) Recommendations: The BCP cement grout equipped in this experiment has significant economic and environmental characteristics compared to pure cement grout, which is consistent with sustainable development strategies. Construction professionals or policymakers should encourage and promote the use of new materials in terms of policy, which has significant strategic significance in solving the technical problems of goaf reinforcement and promoting ecological restoration.

## Figures and Tables

**Table 1:** The main physical and mechanical properties of cement.

Indicators	Specific Surface Area m^2^/kg	Setting Time Min		Compressive Strength MPa		Flexural Strength MPa	
		Initial Setting	Final Setting	7 d	28 d	7 d	28 d
results	412	167	233	23	52	4.9	7.3

**Table 2:** Chemical composition of raw materials.

Raw Materials	Chemical Composition (%)								
	CaO	SiO_2_	Al_2_O_3_	MgO	Fe_2_O_3_	SO_3_	K_2_O	Na_2_O	TiO_2_
cement	59.49	19.41	6.49	4.05	3.4	4.33	0.96	0.61	0.5
waste concrete powder	62.76	20.244	4.996	5.511	2.84	0.62	1.121	0.583	0.36
waste brick powder	8.008	60.99	16.379	2.631	5.697	0.344	2.786	1.868	0.778

**Table 3:** Test scheme of composite proportioning for waste brick powder and waste concrete powder.

Group Numbers	Cement Content (%)	Brick Powder Content (%)	Concert Powder Content (%)
WCP70	30	0	70
WCP60WBP10	30	10	60
WCP50WBP20	30	20	50
WCP80	20	0	80
WCP60WBP20	20	20	60
WCP40WBP40	20	40	40
WCP20WBP60	20	60	20
WCP90	10	0	90
WCP80WBP10	10	10	80
WCP60WBP30	10	30	60
WCP40WBP50	10	50	40
WCP20WBP70	10	70	20

## Data Availability

Not applicable.
